# Celiac disease associated membranous nephropathy - a rare cause or coincidence? A case report

**DOI:** 10.4076/1757-1626-2-7018

**Published:** 2009-06-18

**Authors:** Deepali Prasad, Harshit S Khara, Mohit Gupta, Paul Sterman

**Affiliations:** 1 Department of Internal Medicine, Drexel University College of Medicine - Saint Peter's University Hospital254 Easton Avenue, New Brunswick, NJ 08901USA; 2 Department of Internal Medicine and Nephrology, Drexel University College of Medicine - Saint Peter's University Hospital254 Easton Avenue, New Brunswick, NJ 08901USA

## Abstract

**Introduction:**

Celiac disease is associated with a variety of autoimmune diseases such as type 1 diabetes mellitus, autoimmune thyroid disorders, Sjogren's syndrome and IgA nephropathy, however membranous nephropathy is not recognized amongst one of them.

**Case presentation:**

We report a rare case of nephrotic syndrome due to membranous nephropathy in a patient with celiac disease. A 77-years-old male patient presented with uncontrolled hypertension, anemia and acute renal failure. He was diagnosed with celiac disease and membranous nephropathy confirmed by small bowel and renal biopsy. Patient was treated with gluten free diet and immuno-suppressive therapy; however, he died within 2 to 3 months due to myocardial infarction.

**Conclusion:**

The association between celiac disease and nephrotic syndrome is extremely rare. Only two adult patients with celiac disease and membranous nephropathy have been reported in the literature so far. Since the prevalence of celiac disease ranges between 0.75% and 4.54%, the question arises whether the coexistence of celiac disease and membranous nephropathy is just a coincidence or a rare association. As they both are immune mediated diseases, a link between them is a strong possibility.

## Introduction

Celiac disease (CD) is an immune mediated enteropathy, associated with a variety of autoimmune diseases such as type 1 diabetes mellitus, autoimmune thyroid disorders, Sjogren's syndrome and IgA nephropathy amongst others [1]. However, membranous nephropathy is not one of its known associations. We report a rare case of nephrotic syndrome associated with membranous nephropathy in a patient with CD.

## Case presentation

**Figure 1.**
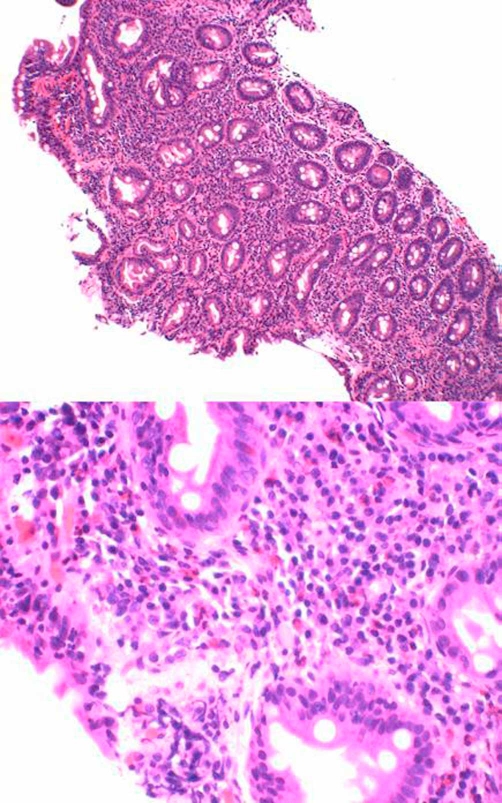
Small intestinal mucosa showing marked chronic inflammation with intraepithelial lymphocytic infiltrate and villous atrophy suggestive of celiac sprue.

A 77-years-old Caucasian male with past medical history of hypertension, iron deficiency anemia and gout was admitted to our hospital with scrotal and bilateral leg swelling along with a blood pressure of 257/117. Labs showed a BUN of 29, serum creatinine of 1.96 and hemoglobin of 10.2. Further testing revealed a low serum albumin of 1.9 and 4.2 gm/day of proteinuria with no RBC casts in urine analysis. Upper GI endoscopy with small bowel biopsy done for evaluation of anemia revealed villous atrophy with intraepithelial lymphocytic infiltrate consistent with celiac disease (Figure 1). Anti-endomysial antibody was positive whereas ANA, C3 and C4 were normal. Hepatitis B and C serologies were also negative. No monoclonal protein was detected in serum and urine immunofixation electrophoresis examination.

**Figure 2.**
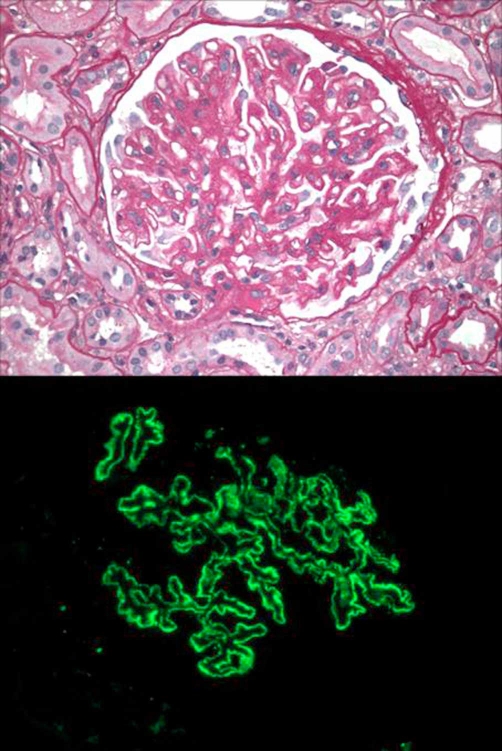
Renal biopsy showing membranous glomerulopathy, tubular atrophy, interstitial fibrosis with interstitial inflammation.

Renal biopsy subsequently revealed stage II-III membranous nephropathy. Immunofluorescence studies showed granular global subepithelial deposits which stained 3+ for IgG, 1+ for IgM/T/IgA, 3+ for C3, +/− for C1, 3+ for kappa and 3+ for lambda; supporting the diagnosis of membranous nephropathy. Electron microscopy showed 2-3+ global subepithelial/T/intramembranous electron dense deposits, 100% foot process effacement, 1-2+ segmental subendothelial fluff and 1+ segmental GBM duplication consistent with membranous nephropathy (Figure 2). Review of patient's medication list did not reveal any drugs causing membranous nephropathy. Patient was put on a gluten free diet and started on amlodipine, hydralazine and clonidine for blood pressure control. He was started on irbesartan to control the proteinuria and furosemide for the edema, along with allopurinol for gout prophylaxis. The patient was initiated on steroids (prednisone 60 mg/day) as well as an additional immunosuppressive agent (cyclophosphamide 75 mg/day). However, within a period of 2 to 3 months of starting therapy, patient died due to myocardial infarction.

## Discussion

The association between CD and nephrotic syndrome is extremely rare. To the best of our knowledge and extensive literature search, we only found two adult patients reported with CD and membranous nephropathy in the past from Europe. The first case report of this association was by Casella et al from Italy, describing a 32 year old man with membranous nephropathy (in remission after 6 months of prednisone), who subsequently received diagnosis of CD (based on serology and duodenal biopsy) and ulcerative colitis [2]. There is another case reported by Halma et al from The Netherlands, in which a 62-years-old male with hypertension, 10 gm/day of proteinuria and anemia due to vitamin B12 and folic acid deficiency had duodenal biopsies and a kidney biopsy which confirmed the diagnosis of CD and membranous nephropathy respectively [3]. However, our case is the first report of this association from the United States.

CD is a much greater problem in the United States than previously anticipated. The prevalence of CD ranges from 0.75% in the not-at-risk subjects to 4.54% among first-degree relatives of patients with CD [4]. The question arises whether the coexistence of CD and membranous nephropathy is just a coincidence or a rare association. Since they both are immune mediated diseases, a link between them is a strong possibility and thus it is important to bring to light this rare probable connection as undiagnosed CD patients may be exposed to the risk of long term complications such as anemia, infertility, osteopenia or cancer (intestinal lymphoma) [5]. Thus it is important to keep this association in mind for primary care physicians, internists, gastroenterologists and nephrologists when working up a patient with anemia and renal failure.

## Abbreviations

BUN: blood urea nitrogen
CD: Celiac disease
GBM: Glomerular basement membranes
